# Physiological and Transcriptomic Responses of Bok Choy to Heat Stress

**DOI:** 10.3390/plants13081093

**Published:** 2024-04-13

**Authors:** Cuina Dong, Xixuan Peng, Xiaona Yang, Chenggang Wang, Lingyun Yuan, Guohu Chen, Xiaoyan Tang, Wenjie Wang, Jianqiang Wu, Shidong Zhu, Xingxue Huang, Jinlong Zhang, Jinfeng Hou

**Affiliations:** 1 Vegetable Genetics and Breeding Laboratory, College of Horticulture, Anhui Agricultural University, 130 West Changjiang Road, Hefei 230036, China; dongcuina@stu.ahau.edu.cn (C.D.);; 2 Provincial Engineering Laboratory for Horticultural Crop Breeding of Anhui, 130 West of Changjiang Road, Hefei 230036, China; 3 Wanjiang Vegetable Industrial Technology Institute, Maanshan 238200, China

**Keywords:** bok choy, thermotolerance, transcriptome analyses, antioxidants, sulfur metabolism

## Abstract

High temperatures have adverse effects on the yield and quality of vegetables. Bok choy, a popular vegetable, shows varying resistance to heat. However, the mechanism underlying the thermotolerance of bok choy remains unclear. In this study, 26 bok choy varieties were identified in screening as being heat-resistant at the seedling stage; at 43 °C, it was possible to observe obvious heat damage in different bok choy varieties. The physiological and biochemical reactions of a heat-tolerant cultivar, Jinmei (J7), and a heat-sensitive cultivar, Sanyueman (S16), were analyzed in terms of the growth index, peroxide, and photosynthetic parameters. The results show that Jinmei has lower relative conductivity, lower peroxide content, and higher total antioxidant capacity after heat stress. We performed transcriptome analysis of the two bok choy varieties under heat stress and normal temperatures. Under heat stress, some key genes involved in sulfur metabolism, glutathione metabolism, and the ribosome pathway were found to be significantly upregulated in the heat-tolerant cultivar. The key genes of each pathway were screened according to their fold-change values. In terms of sulfur metabolism, genes related to protease activity were significantly upregulated. Glutathione synthetase (*GSH2*) in the glutathione metabolism pathway and the *L3e*, *L23*, and *S19* genes in the ribosomal pathway were significantly upregulated in heat-stressed cultivars. These results suggest that the total antioxidant capacity and heat injury repair capacity are higher in Jinmei than in the heat-sensitive variety, which might be related to the specific upregulation of genes in certain metabolic pathways after heat stress.

## 1. Introduction

Bok choy, also known as rape, originated in China, where it is distributed in the north and south. In China today, bok choy is widely planted and has a high yield; its sales rank first among all kinds of vegetables, playing a role in stabilizing market vegetable prices.

Heat stress is one of a number of environmental factors that limit the growth and productivity of crops globally [1,2,3]. Heat stress occurs when the ambient temperature exceeds the temperature suitable for plant growth and development for a prolonged period of time. Heat stress affects the growth performance of plants, delaying vegetative growth [4,5,6]. The results of crop-based model analysis show that a 1 °C increase in the seasonal temperature in tropical and subtropical regions directly corresponds to a 2.5–16% loss in crop yield [7].

From a biochemical point of view, sulfur is of interest as a mineral element that can switch between a variety of existing forms, so sulfur-containing compounds are involved in a variety of chemical reactions. The amino acid cysteine is a product of the sulfate-reductive assimilation pathway. Cysteine is a source of reduced sulfur for many other essential metabolites, including methionine. Glutathione (GSH) thiol residues, plant chelators, and other functional peptides are necessary for detoxifying heavy metals, foreign organisms, pathogen- and insect-borne molecules, and reactive oxygen species (ROS). 3′-phosphoadenosine 5′-phosphosulfate synthetase (PAPS), produced in the sulfate assimilation pathway, provides activated sulfates for the sulfation of metabolites that play a unique role in abiotic and biological stress mitigation [8].

The signal transduction components, transcription factors, and proteins (cysteine and glutathione) associated with stress-induced sulfur metabolism are the main factors in plant responses to high temperatures. The stress response of plants also results in changes in starch content [9]. The identification of heat response genes from studying the appropriate genotypes may provide some insights into the mechanisms of heat resistance. The results of a transcriptomic analysis of two bok choy cultivars revealed a number of genes involved in heat stress, such as those for heat shock proteins (HSPs), membrane leakage genes, and those encoding proteins involved in ROS homeostasis [10]. The expression of genes associated with oxidative stress, protein protection, programmed cell death, biological stress reactions, and metabolism also differs under heat stress [11].

Heat stress accelerates the generation of ROS, including singlet oxygen (^1^O_2_), O_2_^−^, H_2_O_2_, and –OH, inducing oxidative stress in plants [12,13,14]. These reactive oxygen molecules act as signal molecules, triggering plant resistance to abiotic stress. Additionally, accumulated ROS causes irreversible oxidative damage to plants [15,16,17]. The reaction center of photosynthesis and respiration is the main site for the production of ROS, which can attack a variety of biological molecules and cause membrane lipid peroxidation, resulting in cell metabolism disorders [2]. The stability of the cell membrane structure is closely related to the tolerance of plants to high temperatures [18,19,20]. ROS mainly acts on membrane lipid and pigment peroxidation, altering membrane permeability and function [21]. H_2_O_2_ is a toxic compound that is harmful to cells, resulting in lipid peroxidation and membrane injury [22] and consequent production of the highly reactive and cytotoxic aldehyde derivative MDA [23]. Lipid peroxidation has been widely used as an indicator of the damage to membranes caused by free radicals under stress conditions. MDA is one of the most important and widely studied products of peroxidases (PUFAs). MDA is mainly produced in chloroplasts [24].

RNA sequencing (RNA-seq) is a high-throughput sequencing method used to analyze transcriptomes with or without genomic information. It is a useful tool for simultaneously estimating abundance and discovering new functionally significant transcripts [25]. The aims of this study are to (1) determine the physiological and biochemical changes in bok choy under stress, (2) elucidate the role of the sulfur metabolism pathway in resistance to heat stress in bok choy, and (3) provide a theoretical reference for heat-resistant cultivation and breeding.

## 2. Results

### 2.1. Evaluation of Heat Tolerance of Different Bok choy Varieties

**Figure 1:**
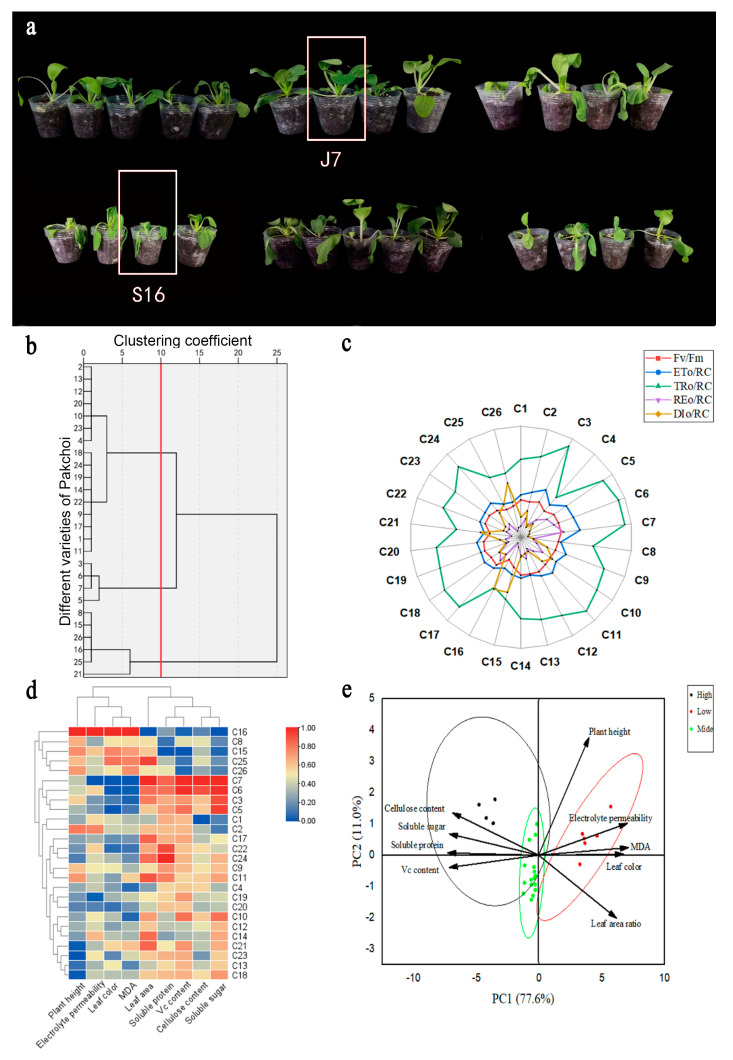
Physiological trait differences in 26 bok choy varieties under heat stress: (**a**) Phenotypic differences at high temperature. Performance differences in the 26 bok choy varieties in an artificial climate under high temperature (day/night; 35/25 °C). From left to right, the first line represents varieties C1–C13, and the second line represents varieties C14–C26. (**b**) Clustering of the heat damage index in the seeding period. (**c**) Radar diagram of the chlorophyll fluorescence parameter. (**d**) Heatmap clustering of all indices at the seedling stage. (**e**) Two-factor principal component analysis for evaluation of all indices at the seedling stage. The above physiological indexes are the results of three biological replicates.

To comprehensively evaluate the heat tolerance of bok choy, we simultaneously treated 26 cultivars in greenhouses and in an artificial climate. In both cases, we set the heat stress temperature (Figure S1). The phenotypes of the different varieties of bok choy differed after experiencing high-temperature stress (Figure 1a). We determined the heat damage index of the 26 bok choy varieties at the stage of 4–5 true leaves (Table S1). Using cluster analysis, the 26 bok choy varieties were classified as heat-tolerant, medium heat-tolerant, and heat-sensitive with a Euclidean distance of 10.00 (Figure 1b). After high-temperature treatment, in terms of the chlorophyll fluorescence characteristics of bok choy in the seedling stage compared with S16, the ratio of variable fluorescence to maximum fluorescence (Fv/Fm), the rate of electron transport flux of the photosystem II reaction center (ETo/RC), the energy flux trapped by one active photosystem II reaction center (TRo/RC), and transport to the PSI terminal (REo/RC) of J7 were higher by 49.07%, 61.11%, 77.31%, and 300.91%, respectively, and the level of antenna Chl (DIo/RC) decreased by 80.3% (Figure 1c). The heat-tolerant category included cultivars C3, C5, C6, and C7, and the heat damage index was about 20%; the heat-sensitive cultivars were C8, C15, C16, C21, C25, and C26, and the heat damage index was about 50%; the rest were medium heat-tolerant cultivars, and the heat damage index was between 30% and 40%.

Physiological indexes related to the heat resistance of bok choy were measured, including agronomic traits of bok choy at the seedling stage under high temperatures (Table S2) and physiological and biochemical indexes (Table S3). The recorded agronomic traits included the leaf area ratio, plant height, and color difference. The recorded physiological and biochemical indices include soluble protein, soluble sugar, Vc, malondialdehyde (MDA), relative conductivity, and cellulose content. Heatmap clustering and principal component analysis were performed using these indicators (Figure 1d,e), and the results were in agreement with those of the clustering shown in Figure 1c. Based on the analysis of growth conditions and phenotype, the heat-tolerant variety J7 (C7) and the heat-sensitive variety S16 (C16) were selected for use in the following experiments.

### 2.2. Agronomic Characteristics under Heat Stress

Heat stress resulted in differing effects on the growth of different bok choy varieties, as indicated in Table 1. Compared with the control group not subjected to heat stress, J7-CK, the dry shoot weight, fresh shoot weight, leaf area, and plant height of J7 under heat stress decreased by 24%, 23%, 12%, and 13%, respectively, while the leaf color increased by 49%. Heat stress had a greater impact on S16 than on J7: compared with the non-HS control (S16-CK), the dry shoot weight, fresh shoot weight, leaf area, and plant height of S16 significantly decreased by 37%, 35%, 28%, and 24%, respectively, while leaf color increased by 39%.

### 2.3. Physiological and Biochemical Indices under Heat Stress

**Figure 2:**
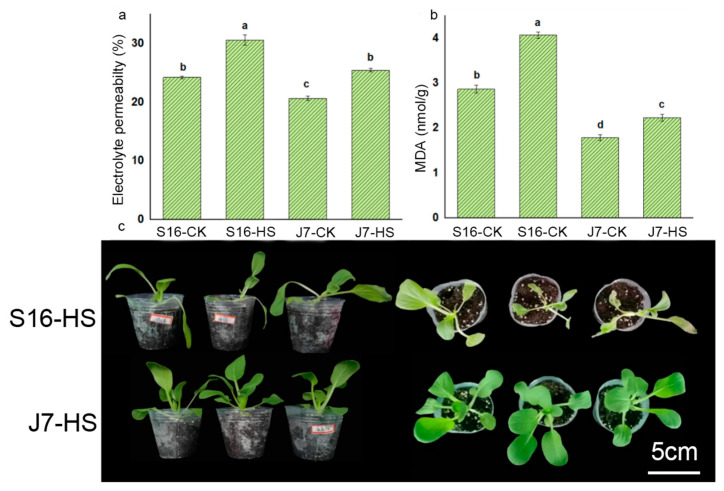
The influence of heat on electrolyte permeability, MDA, and the growth of J7 and S16: (**a**) electrolyte permeability; (**b**) MDA; (**c**) growth condition of whole plants after being treated at 43 °C for 2 days. S16-HS: S16 treated at 43 °C for 24 h; J7-HS: J7 treated at 43 °C for 24 h. All data at 43 °C were determined following a 24 h treatment. Data are mean ± SD, and the difference between the letters (a–d) in Duncan’s test indicates a significant difference at *p* < 0.05.

Malondialdehyde (MDA), as one of the important heat resistance indices, was significantly influenced by heat treatment. The MDA content of S16 and J7 increased by 29.73% and 19.73%, respectively, at 43 °C compared with the corresponding CK controls (Figure 2b). Likewise, heat stress increased electrolyte permeability (Figure 2a) by 18.89% for J7 and by 20.83% for S16 compared with the controls. The growth characteristics of both varieties are shown in Figure 2c. These results indicate that S16 is more damaged by heat stress than J7. 

**Figure 3:**
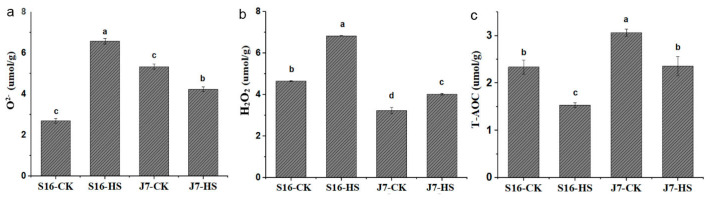
(**a**) O_2_^−^; (**b**) H_2_O_2_; (**c**) T-AOC. S16-HS: S16 treated at 43 °C for 24 h; J7-CK: J7 treated at 35/25 °C. All data were determined at 43 °C for 24 h. Data are mean ± SD, and different letters (a–d) indicate a significant difference (*p* < 0.05) per Duncan’s test.

Heat stress was also found to affect antioxidant capacity. The content of ROS increased under heat stress to different extents in the two cultivars. The higher the antioxidant capacity, the greater the heat resistance. Under heat stress, the total antioxidant capacity (T-AOC) of J7 decreased by 22.93% in comparison with the control (Figure 3c), whereas that of S16 decreased by 34.35%. Under this condition, O_2_^−^ and H_2_O_2_ contents increased by 59.02% and 31.84% in S16, respectively, and by 23.59% and 19.53% in J7, respectively, compared with the corresponding control (Figure 3a,b). The increases in J7 were much lower than those in S16. 

### 2.4. Identification of Differentially Expressed Genes by Transcriptome Sequencing

The bok choy transcriptome was analyzed to explore the genetic response to heat treatment. J7 and S16 were exposed to 43 °C for 24 h. Leaves were harvested, and samples were sequenced in triplicate based on separate RNA samples. The four groups each contained 12 samples for RNA sequencing. We obtained over 563.63 million original reads and 561.87 million clean reads from these samples (Table S4). Moreover, the percentage of clean reads was over 87.48%. These results indicate that the sequencing output and read quality are sufficient for further analysis.

### 2.5. Identification and Enrichment Analysis of DEGs

**Figure 4:**
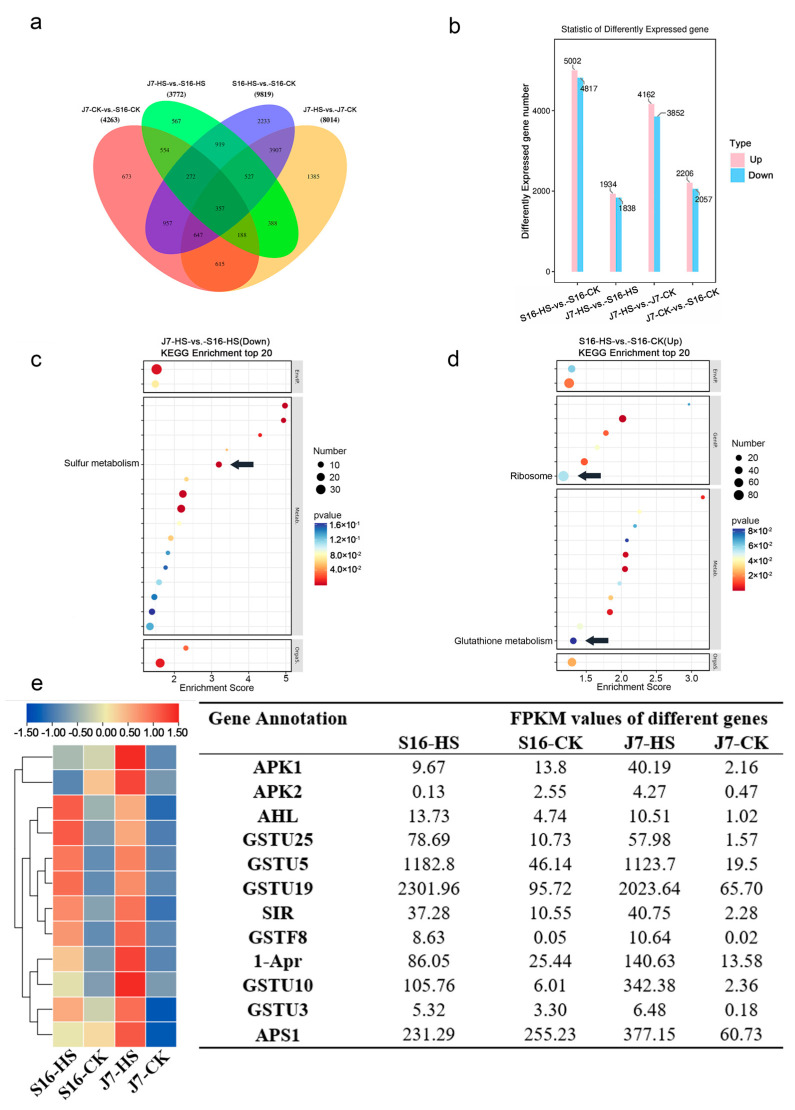
Expression of transcription factors in different varieties of bok choy under heat stress: (**a**) Venn diagrams of DEGs; (**b**) up- and downregulated DEGs; (**c**,**d**) KEGG analysis of J7-HS-vs.-S16-HS (down) and S16-HS-vs.-S16-CK (up). The arrows in the figure indicate the key pathways; (**e**) heatmap.

Altogether, 14,189 DEGs were found in the transcriptome. These DEGs were grouped into 15 discrete subgroups, among which 56.48% (8014/14,189), 30.04% (4263/14,189), 26.58% (3772/14,189), and 69.20% (9819/14,189) were group-specific DEGs in J7-HS-vs.-J7-CK, J7-CK-vs.-S16-CK, J7-HS-vs.-S16-HS, and S16-HS-vs.-S16-CK, respectively. The Venn diagram of the 14,189 unique DEGs in the four groups is shown in Figure 4a. 

The DEGs were categorized into 52 GO terms in the four different groups in the heat treatments (Figure S3). The top five significantly enriched pathways in the four different groups were 46: Metabolic process (1190~5235); 47: Cellular process (1236~5058); 49: Cellular process (1507~6597); 51: Cell part (1890~8118); and 52: Cell (1895~8133). The top five significantly enriched pathways in the J7-HS-vs.-J7-CK group were 1: Channel regulator activity (5665); 13: Electron carrier activity (4337); 22: Symplast (6623); 49: Cellular process (6597); 51: Cell part (8118); and 52: Cell (8133), which were significantly more enriched than the other three groups. The top three significantly enriched pathways in J7-HS-vs.-J7-CK and S16-HS-vs.-S16-CK were metabolism (507, 356), translation (434, 241), and folding, sorting, and degradation (368, 257) (Figure S4). Gene enrichment was more significant for J7-HS-vs.-J7-CK and S16-Hs-vs.-S16-CK than for J7-HS-vs.-S16-HS and J7-CK-vs.-S16-CK.

Sulfur metabolism in heat-tolerant varieties was one of the top 20 enriched metabolic pathways, but its enrichment fraction and the number of differential genes in heat-resistant varieties were higher than those in heat-sensitive varieties. Most sulfur metabolism occurs in chloroplasts and is associated with the stress-induced accumulation of ROS. This result is consistent with the low O_2_^−^ and H_2_O_2_ in the J7 variety. Combined with the above physiological and biochemical indices, the analysis mainly focused on the sulfur metabolism and glutathione metabolism pathways, which are related to ROS, and the ribosome metabolism pathway, which is related to recovery after stress injury (Figure 4c,d).

To study the molecular mechanism of the bok choy response to heat stress, the functions of the genes in pathways related to sulfur metabolism, ribosome metabolism, and glutathione metabolism were further explored. According to the fold-change value, genes with the four highest fold-change values were selected from each metabolic pathway (Table S5). According to the FPKM values of these genes, the genes were classified, and a clustered heatmap was constructed (Figure 4e).

### 2.6. DEGs of Sulfur Metabolism Pathway

**Figure 5:**
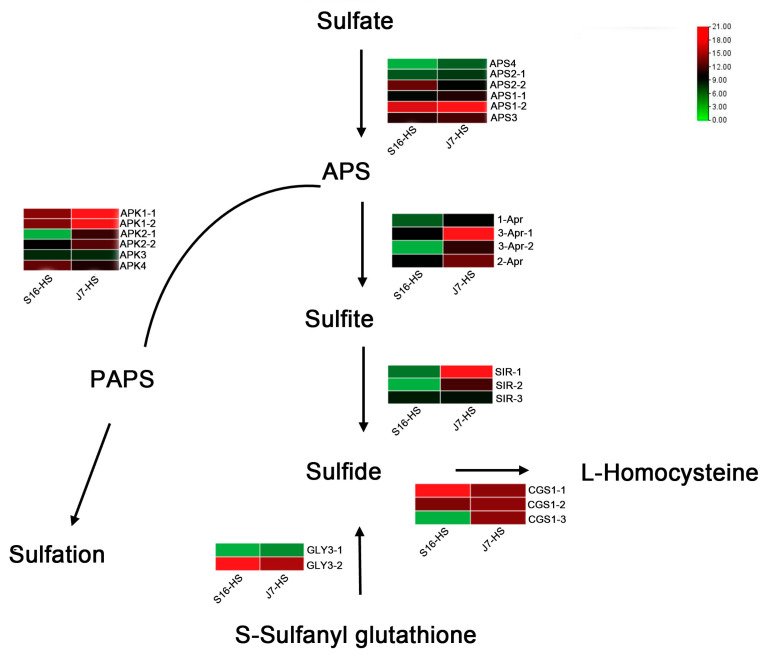
Heatmap of DEGs related to sulfur metabolism. DEGs were screened in J7-HS-vs.-J7-CK, with J7-CK as the control. APS, ATP sulfurylase; APK, adenylyl-sulfate kinase; 1-Apr, 5′-adenylylsulfate reductase; SIR, assimilatory sulfite reductase (ferredoxin); GLY3, persulfide dioxygenase; ETHE1, homolog; CGS1, cystathionine gamma-synthase 1.

The three key enzymes involved in sulfur metabolism are 3′-phosphoadenosine 5′-phosphosulfate synthetase (PAPS), cystatin C (CysC), and sulfite reductase (SiR). Interruption of these enzyme reactions can result in changes in ROS content. Thus, we analyzed the expression of the genes encoding the enzymes involved in this metabolic pathway. According to the transcriptome sequencing results, the 22 key genes in the sulfur metabolism pathway of the two bok choy cultivars before and after heat stress were compared and analyzed. The expression of *APS1* of the two varieties in the *APS*-mediated conversion to sulfate was significantly upregulated, whereas the expression of *APS4* showed a downward trend. The expression of *3-Apr*, encoding a protein involved in the *APS*-mediated process of conversion to sulfite, was significantly upregulated in both cultivars, and *APK1* expression was significantly upregulated in the PAPS pathway. Upregulation of *SIR* expression was noted in the process of conversion from sulfite to sulfide. The gene *GLY3* plays a major role in the process of S-sulfonyl glutathione conversion to sulfide. The expression of *CGS1*, resulting in a product involved in the conversion from sulfide to homocysteine, showed the opposite trend; it was significantly upregulated in heat-resistant varieties but not in heat-sensitive varieties (Figure 5).

### 2.7. DEGs of Glutathione Metabolism Pathway

**Figure 6:**
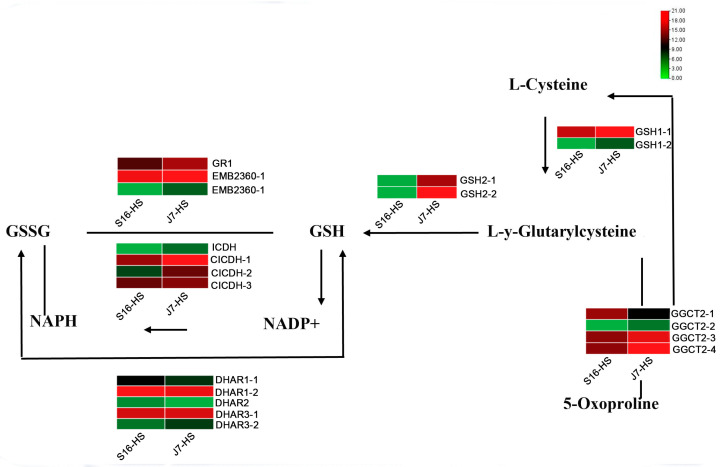
Heatmap of DEGs related to glutathione metabolism. DEGs were screened in J7-HS-vs.-J7-CK, with J7-CK as the control. GR1, glutathione reductase; GSH1, glutamate–cysteine ligase; GSH2, glutathione synthetase; ICDH, peroxisomal isocitrate dehydrogenase [NADP]; CICDH, cytosolic isocitrate dehydrogenase [NADP]; DHAR, glutathione S-transferase DHAR; GGCT2, gamma-glutamylcyclotransferase 2.

From the analysis of key genes in the glutathione metabolic pathway, we found that during the conversion of L-Y-glutarylcysteine to GSH, the expression of *GSH2* was significantly upregulated in heat-resistant varieties after heat stress, whereas the opposite was observed in heat-sensitive varieties. After heat stress, the expression levels of *CICDH*, *DHAR3*, and *EMB2360* in the process of NAPH conversion to NADP+ and GSH to GSSG were upregulated, indicating that the glutathione pathway is responsive to heat stress and plays a role in the heat resistance of these cultivars (Figure 6).

### 2.8. DEGs of Ribosome Pathway

**Figure 7:**
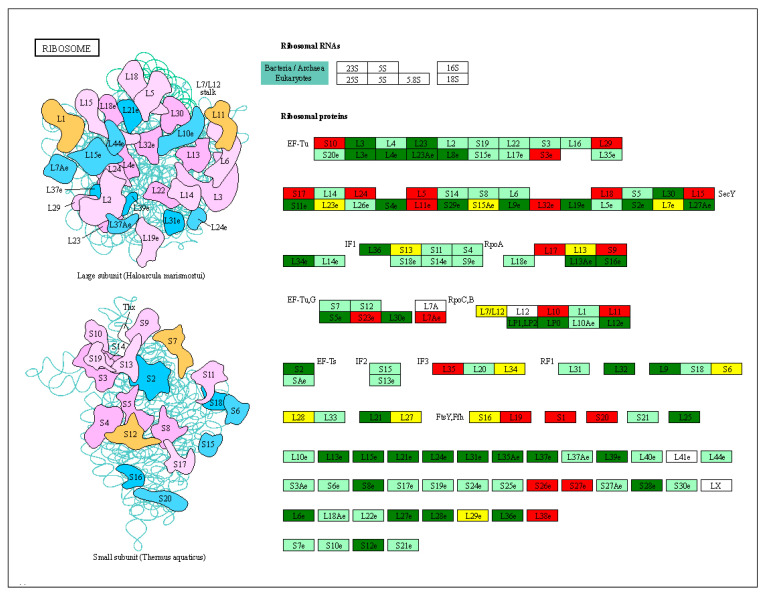
Ribosomal pathway analysis. DEGs were screened in J7-HS-vs.-J7-CK, with J7-CK as the control. L represents the large subunit, and S represents the small subunit. On the subunit diagram, each color represents a class of subunits. In the picture on the right, red represents upregulated genes, green represents downregulated genes, white indicates successful annotation of no differential genes, and yellow indicates no differential genes.

We compared the downregulation of key genes in the small and large subunits in the ribosomal pathway. Ef-Tu (elongation factor thermo unstable) plays a major role in the ribosome translation of proteins. The expression levels of *L3e*, *L4e*, *L23*, *L23Ae*, *L8e*, *S19*, *L17e*, and *L35* in EF-Tu were significantly upregulated in the heat-resistant varieties. We found that 69 ribosomal subunits were upregulated and 42 were downregulated in J7-HS (Figure 7). In addition, 23 small and 46 large subunits were upregulated, and 24 small subunits and 18 large subunits were downregulated. These results suggest that the ribosome pathway plays an important role in protein translation and affects protein production during heat stress.

### 2.9. Experimental Validation

**Figure 8:**
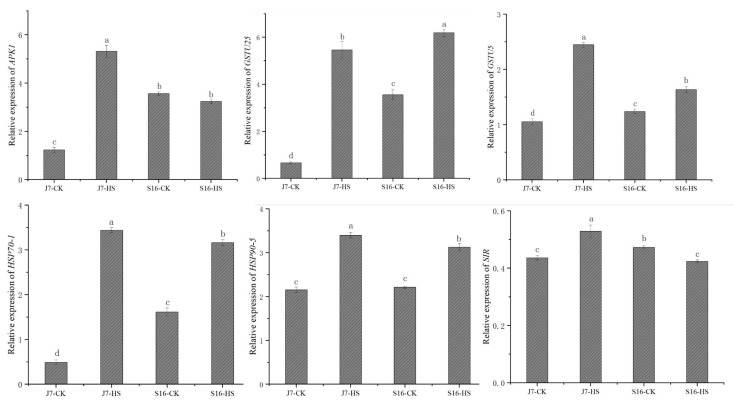
Validation of DEGs by qRT-PCR. Six DEGs were randomly selected, and their transcript levels under both normal temperature and heat-stress conditions were analyzed using qRT-PCR analysis. J7-CK, normal temperature; J7-HS, heat stress; S16-CK, normal temperature; S16-HS, heat stress; line graphs represent qRT-PCR data. Data are presented as the mean ± SD of three biological replicates, the letters a–d represent data significance differences.

To verify the reliability of our transcriptome data, we randomly selected six genes for validation by quantitative real-time PCR (qRT-PCR). We found differences in the expressions of four of the selected genes between the two cultivars under heat stress. After heat stress, more genes exhibited changes in expression in J7 than in S16. The qRT-PCR results showed that the relative expression trends of these six genes are highly consistent with their transcript trends according to RNA-seq. The DEGs are *APK1*, *SIR*, *GSTU25*, *GSTU5*, *HSP70-1*, and *HSP90-5*. The results of both the RNA-seq and qRT-PCR assays showed similar expression patterns for these DEGs, indicating that our RNA-seq data are accurate and reliable (Figure 8).

## 3. Discussion

The morphological signs of heat stress include sunburn on leaves, twigs, branches, and stems; leaf senescence and abscission; shoot and root growth inhibition; and fruit discoloration and damage. Of the varieties, J7 and S16 were chosen in this study because of the differences in their tolerance to heat stress, which allowed us to explore the mechanisms underlying heat tolerance by comparing the two cultivars. Under heat stress, the fresh weight and plant height of J7 were higher than for S16, but the difference was not significant. The chromatic aberration ∆E value and leaf area were lower in J7 than in S16. Under heat stress, only the chromatic aberration value increased, while the other indices decreased. In an earlier study on mung bean, damage to the leaf tips and margins, rolling and drying of the leaves, and necrosis were observed due to heat stress [26]. Although heat stress significantly inhibited biomass accumulation in both varieties in this study, the fresh shoot weight and plant height decreased more in the heat-sensitive S16 than in the heat-tolerant J7. This result suggests that the heat tolerance of bok choy may be improved by regulating plant architecture and leaf shape. Generally, S16 was more severely injured than J7. Wheat [27] species have been observed to significantly differ in heat tolerance due to their genetic makeup. We think that J7 may have some resistance mechanisms that promote its high heat tolerance.

Chlorophyll fluorescence parameters, especially Fv and Fv/Fm, are more suitable than cell membrane thermostability (CMT) for screening bok choy lines for heat tolerance [28]. In the current study, heat stress had differing effects on the morphology and photosynthesis of both varieties. The results indicate that the heat tolerance of bok choy is genotype-specific, which is in line with the findings reported by Mishra [29].

The accumulation of soluble proteins is closely related to the heat resistance of plants [30]. Under high-temperature stress, the soluble protein content decreased to a lesser degree in the bok choy varieties with higher heat resistance than those with less heat resistance. The soluble sugar content of spinach increased after high-temperature treatment [31]. Soluble sugars and soluble proteins are compatible osmolytes, and their accumulation significantly contributes to enhanced plant heat stress tolerance [32,33].

High temperatures damage the structure of the cell membrane and alter the transmembrane transport of ions, water, and organic solutes, thus affecting the function of the membrane [34]. High temperatures can increase cell membrane permeability, thereby increasing electrical conductivity with tissue extravasation. Therefore, the thermal stability of the cytoplasmic membrane is an important index of heat resistance. In plants, MDA is the final product of membrane lipid peroxidation, and its content is an important indicator of the degree of membrane lipid peroxidation [35]. The results of Fv/Fm, relative conductivity (REC), and MDA content indicate the close relationship of these parameters with heat tolerance, so these indices may be used as physiological characteristics to identify heat-tolerant genotypes of bok choy and other plants.

Ascorbic acid (AsA) and reduced GSH are potent nonenzymatic antioxidants within the cell [36]. AsA removes the most hazardous forms of ROS, namely hydroxyl radicals (–OH), superoxide anions (O_2_^−^), H_2_O_2_ (via APX), and glutathione, which are involved in maintaining the Halliwell–Asada pathway in a reduced state and serve as the main thiol–disulfide redox buffer in plants. A correlation exists between tolerance and antioxidant levels in wheat varieties [37]. In the present study, genes in the glutathione pathway were significantly upregulated in J7 compared with S16. The higher T-AOC in J7 partly protected the thylakoid membrane system from oxidative damage (declining MDA and H_2_O_2_ contents).

To determine the relationship between the differences in ROS content and the heat resistance of the two varieties, the experimental results were analyzed in combination with the transcriptomic results. Under stress, the accumulation of ROS can effectively promote the expression of transcription factors, further regulating the expression of stress-responsive genes and improving plant resistance [38]. Excessive ROS reacts with phospholipids and membrane receptor proteins in lipid peroxidation [39]. Excessive ROS can also lead to cellular structure destruction, including chromatin condensation, chloroplast destruction, mitochondrial swelling, and DNA replication [40,41], which ultimately lead to programmed cell death. Raja et al. [42] reported that ROS coupling with Ca^2+^ and electrical signals can form a tolerance mechanism that rapidly transmits signals and activates adjacent and distal cells.

A number of biological and molecular functions are affected by temperature stress [43]. Temperature stress has a substantial impact on the chemistry and physics of biological systems [44]. Temperature stress can affect several properties of the structural components and biomolecules of cells, including the activity, folding, stability, and assembly of proteins [45], the structure of lipids [46,47], and the fluidity of cell membranes [48]. These changes are facilitated via the transcriptional regulation of genes [49,50].

We found more DEGs in J7-HS-vs.-J7-CK and S16-HS-vs.-S16-CK than in J7-HS-vs.-S16-HS and J7-CK-vs.-S16-CK, indicating that the number of DEGs increased in both varieties under heat stress (Figure S2). In addition, more changes occurred in the genes of J7, and the upregulated DEGs of the heat-resistant varieties were found to be more widely distributed, which may underlie J7 having higher stress tolerance than S16.

## 4. Materials and Methods

### 4.1. Plant Materials and Experimental Design

We bought 26 bok choy varieties from the seed market; these cultivars are widely planted in the Yangtze–Huaihe river basin in China. The experiment was conducted in the breeding basement of Wanjiang Vegetable Research Institute, Maanshan, China (31°45′20.4156″ N,118°24′8.3664″ E).

Before heat treatment, we monitored the real temperature for over 30 days in the greenhouse of bok choy cultivation in the summer. The temperature was lowest at approximately 6:00 every day, with an average of 24.5 °C, and the temperature was highest at approximately 14:00, with an average of 44 °C. The temperature from 7:00 to 18:00 was not lower than 35 °C. The 26 bok choy varieties were planted in the artificial climate chamber to select the more heat-tolerant varieties on the basis of the heat-tolerance index. The temperature in the artificial climate chamber was set to 25/18 °C (day/night) for normal temperature and 35/25 °C (day/night) for the high-temperature condition, with 70–75% relative humidity, a light intensity of 9000 lx, and 16 h of light and 8 h of darkness per day. The matrix we used for rearing the plants was turfy soil/perlite = 1:1, and the amount and time of watering were strictly controlled throughout the whole growth process. Samples were taken once every 5 days from 26 bok choy varieties with 5–6 leaves to study the heat resistance of bok choy. The following methods (4.2–4.9) are all indicators of phenotypic screening.

Based on the results of the above experiments and phenotypic screening, further studies on relevant indices and transcriptome analysis were carried out using Jinmei (J7, a heat-tolerant material) and Sanyueman (S16, a heat-sensitive material). J7 and S16 were planted in the artificial climatic chamber at 25/18 °C (day/night). After 30 days, the high-temperature group was subjected to 43 °C heat stress for 24 h, and the third fully expanded functional leaf was collected from the center of the plant every 4 h. Samples were immediately frozen in liquid nitrogen and stored at −80 °C until use in physiological determination and RNA extraction.

### 4.2. Growth Parameters

Plant height was measured using a ruler from the ground to the top of the plant. The fresh weight was gravimetrically measured.

To determine the leaf area ratio, we selected the third leaf counting from the bottom and measured the leaf area with a leaf area meter (Zhejiang Top Cloud-agri Technology Co., Ltd., Hangzhou, China). First, the crimped leaves after heat injury were scanned and denoted as S1, and then the scanned leaf area of flattened leaves was denoted as S2, with the leaf area ratio = (S1/S2) × 100% used to judge the degree of crimping under high temperatures.

To determine chromatic aberration, a portable color difference meter (CR-10 Plus, Hangzhou Kefeng Instrument Co., Ltd., Hangzhou, China) was used. The ΔE values of the middle leaves of bok choy were measured three times, and three samples were measured each time. ΔE is a description of abnormal phenomena such as yellow, white, black, less yellow, and less green. The larger the general deviation of ΔE, the more serious the damage to the plant leaf color by heat stress.

### 4.3. Heat Injury Index (HII)

The morphological degree of change in the plants under heat stress for 30 days was assessed with the heat injury index as follows:

0 = plant grew normally;

1 = 1/4 of leaves showed wilting;

2 = 1/4–1/2 of leaves wilted or turned yellow;

3 = 1/2–3/4 of leaves wilted or turned yellow;

4 = more than 3/4 of leaves wilted or turned yellow;

5 = plant withered.

Heat injury index (%) = Σ(the number of plants of each level × level)/the highest level × the total number of plants of treatment × 100 [51].

### 4.4. Chl Fluorescence

Chl fluorescence was measured using a portable fluorometer (Handy PEA, Hansatech Instruments Ltd., Norfolk, UK) at a wavelength of 650 nm. The fourth fully expanded leaf was dark-adapted for 30 min before measurement. These were measured 30 days after heat treatment, and three fully expanded functional leaves were chosen from the bottom to the top. The following parameters refer to time 0 (Fo): TRo/RC, ETo/RC, REo/RC, and DIo/RC.

### 4.5. Relative Conductivity (REC)

The third functional leaf of each plant was selected to avoid the main vein of the leaf, and a hole punch with a radius of 7.5 mm was used to punch holes in the leaves. We obtained 20 round slices, which we placed in a test tube with 20 mL of deionized water. After standing for 30 min at 25 °C, a Thermo Orion STARA-HB conductivity meter (Thermo Orion, Waltham, MA, USA) was used to determine the conductivity L_0_ of deionized water and the conductivity L_1_ at this time. Then, the test tube was placed in boiling water for 10 min, and the conductivity L_2_ was measured after cooling according to the formula for relative conductivity = (L_1_ − L_0_)/(L_2_ − L_0_) × 100%.

### 4.6. Malondialdehyde (MDA)

Lipid peroxidation was evaluated by estimating the MDA content. The MDA content was determined according to Stewart and Bewley [52], and the calculation formula was:

C (μmmol^−1^) MDA = [6.45 × (D_532_ − D_600_) − 0.56D_450_] × V/m,

where 532, 600, and 450 refer to the absorbance wavelengths; V is the volume of the extracted solution; and m is the mass of the leaf sample.

### 4.7. Soluble Protein, Soluble Sugar, and Vitamin C Content

Soluble protein content was determined by the Bradford method [53]. Using buffer and protein as a contrast, the protein standard curve was drawn. Soluble sugar content was determined by the anthrone method [54]. The absorbance of 625 nm was determined using glucose as a standard. The content of vitamin C was determined by the titrimetric method with 2,6-dichloroindophenol. The VC content was determined according to the volume of dye consumed.

### 4.8. Cellulose

The cellulose fraction was obtained using a method previously described by Yoon et al. [55] with slight modifications.

### 4.9. H_2_O_2_, O_2_^−^, and T-AOC

H_2_O_2_, O_2_^−^, and total antioxidant capacity (T-AOC) were determined using Solarbio kits (BC 3590, BC 1290, and BC1310, Solarbio, Beijing, China).

### 4.10. Transcriptomic Data Analysis

A mirVana™ miRNA Isolation Kit (Ambion, Austin, TX, USA) was used to extract the total RNA of 12 samples from the four treatments, according to the manufacturer’s instructions. An Agilent 2100 Bioanalyzer (Agilent Technologies, Santa Clara, CA, USA) was used to assess RNA integrity. Samples with RNA integrity (RIN) ≥ 7 were analyzed. Libraries were constructed using a TruSeq Stranded mRNA LT Sample Prep Kit (Illumina, San Diego, CA, USA), following the manufacturer’s instructions. Then, the libraries were sequenced on an Illumina sequencing platform (HiSeq TM 2500 or Illumina HiSeq X Ten), and 125 or 150 bp paired-end reads were generated. After quality inspection, an Illumina sequencer was used for sequencing. To achieve high-quality readings that could be used for further analysis, we performed a quality check using Trimmomatic [56]; joints, poor-quality bases, and n bases were removed. The clean, filtered reads were compared with the reference genome of B. rapa with HISAT2 [57]. The gene fragments per kilobase of transcript per million mapped reads (FPKM) values were quantified with Cufflinks v0.9.3 software [58,59]. HtSeq counts were used to determine gene expression differences and calculate the number of reads of the genes in each sample [60]. The data were normalized with an estimated size factor function using the “DESeq” package in R. The “nbinom Test” function was used to calculate the fold change and *p*-values. The false discovery rate (FDR) was used to account for multiple comparisons. DEGs with a *p*-value < 0.05 and |log_2_ (fold change)| > 1 were chosen. GO and KEGG enrichment analyses were performed on the DEGs to identify biological functions or pathways that were primarily influenced by the DEGs. Then, the DEGs were clustered via unsupervised hierarchical clustering, and their expression patterns in different samples were presented in the form of heatmaps.

### 4.11. Validation of RNA-Seq by qRT-PCR

Based on the results of transcriptome analysis, six candidate genes were selected, the corresponding nucleotide sequences were searched in the transcriptome, and the selected DEGs were verified by qRT-PCR. The total RNA was isolated from S16-CK, S16-HS, J7-CK, and J7-HS using a total RNA kit (Takara Biomedical Technology Co., Beijing, China). Primer 6 software was used to design specific primers for the six genes identified from the transcriptomic data. The qRT-PCR primers designed for the selected DEGs genes are listed in Table S6; the gene encoding actin was used as an internal reference gene. qRT-PCR was performed using SYBR GREEN Master Mix (Vazyme Biotechnology Co., Ltd., Nanjing, China). Relative gene expression levels were calculated using the 2^−ΔΔCT^ method [61].

### 4.12. Statistical Analysis

In this study, plants were randomly sampled. Data are presented as the mean ± SD for three biological replicates. SPSS v22.0 (SPSS Institute Inc., Chicago, IL, USA) was used for the statistical analyses. Tukey’s post hoc test was used for mean comparisons using a *p*-value < 0.05. Bar charts were created with Origin 2019b 64-bit software.

## 5. Conclusions

**Figure 9:**
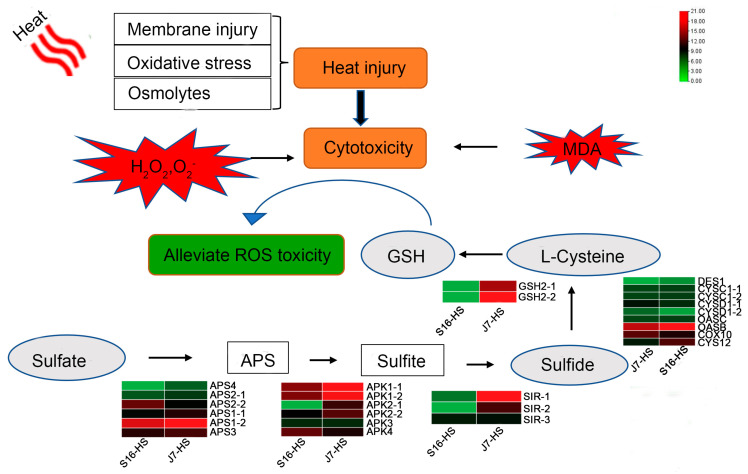
Proposed model of the heat stress response of J7 and S16. Heat stress increases permeability, damages the membrane, and causes oxidative stress. ROS and MDA contents are elevated, along with cell toxicity. The upregulation of genes related to pathways of sulfur and glutathione metabolism and L-cysteine, linking the two metabolites, is associated with decreasing the toxicity of ROS and thus increasing the heat tolerance of the plants.

In this study, a high-temperature treatment to induce heat stress in bok choy was simultaneously applied in a greenhouse and artificial climate chambers. We classified the heat tolerance of 26 cultivars and found that the heat injury index can be conveniently used to evaluate heat tolerance in bok choy at the seedling stage. We also selected a heat-resistant variety, C7 (J7), and a heat-sensitive variety, C16 (S16), for a follow-up study, where we compared and analyzed the physiological morphology and indicators of the J7 and S16 cultivars under heat stress and found that ROS play a significant role in the heat tolerance of bok choy. Next, based on the analysis of the transcriptomic dataset, we succeeded in identifying several pathways associated with ROS (Figure 9). This study was helpful for elucidating the molecular mechanism underlying the growth and development of bok choy, thus providing a basis for breeding heat-resistant varieties of bok choy.

## Figures and Tables

**Table 1:** Effect of heat on the growth parameters of J7 and S16.

Treatment	Dry Shoot wt (g)	Fresh Shoot wt (g)	Leaf Area (cm^2^)	Leaf Color (ΔE)	Plant ht (cm)
S16-CK	0.22 ± 0.13 b	2.34 ± 0.15 b	6.81 ± 0.15 b	5.803 ± 0.17 b	7.27 ± 0.39 b
S16-HS	0.14 ± 0.02 c	1.53 ± 0.05 c	4.87 ± 1.01 c	8.06 ± 0.45 a	5.53 ± 0.45 c
J7-CK	0.29 ± 0.05 a	3.06 ± 0.08 a	10.09 ± 0.76 a	3.54 ± 0.11 c	9.43 ± 0.53 a
J7-HS	0.22 ± 0.15 b	2.36 ± 0.20 b	8.93 ± 0.46 a	5.27 ± 0.02 b	8.17 ± 0.21 b

S16-CK (Sanyueman + 25/18 °C), S16-HS (Sanyueman + 35/25 °C + 30 d + 43 °C + 24 h), J7-CK (Jinmei + 25/18 °C + 31 d), and J7-HS (Jinmei + 35/25 °C + 30 d + 43 °C + 24 h). All data correspond to determination after growth for 30 days. Data are mean ± SD, and different letters (a–c) indicate a significant difference at *p* < 0.05 per Duncan’s test.

## Data Availability

The datasets presented in this study can be found in online repositories. The name of the repository and accession number can be found below: National Center for Biotechnology Information (NCBI) BioProject, PRJNA1080307.

## Supplementary Materials

The following supporting information can be downloaded at: https://www.mdpi.com/article/10.3390/plants13081093/s1, Table S1: The index of heat damage of different genotypes of baby bok choy at the seeding stage under heat stress; Table S2: Agronomic traits of 26 varieties at high temperatures; Table S3: Physiological and biochemical indexes of 26 varieties; Table S4: Sequencing data for 12 libraries were obtained by RNA sequencing; Table S5: List of differentially expressed genes associated with interested pathways under heat stress; Table S6: Genes and primers for qRT-PCR analysis; Figure S1: Daily maximum and minimum temperatures change within 30 days of the greenhouse; Figure S2: Volcano plots for expressed genes in the four comparison groups; Figure S3. GO enrichment diagram; Figure S4. KEGG enrichment diagram.
