# Supplementary material for: Physiological and Transcriptomic Responses of Bok Choy to Heat Stress
Source: Plants (Basel). 2024 Apr 13;13(8):1093. doi: 10.3390/plants13081093 (PMC11053463; doi:10.3390/plants13081093)
Supplement: Supplementary file 1 [file plants-13-01093-s001.zip › plants-2914143-supplementary.pdf]

Table S1. The index of heat damage of different genotypes of baby bok choy at the seeding stage under heat stress.

Material Code	Heat Damage	Variety Name	Material Code	Heat Damage	Variety Name
C1	0.36	Aiguanjun	C14	0.38	Yuanzhongshanghaiqi ng
C2	0.31	Jimaocai	C15	0.55	Shanghaikangre105
C3	0.18	Xiaxin	C16	0.56	Sanyueman
C4	0.27	Aijiaohuang	C17	0.35	Pinweiyixia
C5	0.23	Jinpin	C18	0.39	Zhongxia
C6	0.18	Degaoxiaxing	C19	0.39	Yulonghuaxiu
C7	0.16	Jinmei1	C20	0.3	Huawangqinggengcai
C8	0.55	Qibaoqingcai	C21	0.44	Degaoqingxia
C9	0.35	Zhonggengbai	C22	0.37	Dianmei
C10	0.32	Liqunqinggeng	C23	0.31	Xiasuqing
C11	0.33	Gaogengbai	C24	0.39	Zajiaoshanghaiqing
C12	0.3	Huayouqinggeng bai	C25	0.52	Siyueman
C13	0.31	Xiadi	C26	0.54	Wuyueman

Table S2. Agronomic traits of 26 varieties at high temperatures.

Material Code	Leaf Area Ratio (%)	Plant Height (cm)	Leaf Color (ΔE)
C1	82.77 ± 0.01 de	6.53 ± 1.24 efg	3.22 ± 0.08 ab
C3	91.75 ± 0.03 ab	4.30 ± 0.16 g	2.09 ± 0.15 e
C4	91.63 ± 0.03 b	5.07 ± 0.97 g	4.32 ± 0.08 ab
C5	92.29 ± 0.01 ab	4.97 ± 0.12 g	2.32 ± 0.47 j
C6	94.38 ± 0.01 a	4.17 ± 0.12 g	2.73 ± 0.08 fgh
C7	95.10 ± 0.02 a	5.57 ± 0.25 g	2.15 ± 0.06 fghi
C8	82.75 ± 0.02 de	9.07 ± 1.76 abcde	7.61 ± 0.46 b
C9	86.90 ± 0.03 c	6.87 ± 2.41 cdefg	3.30 ± 0.03 c
C10	85.24 ± 0.01 cd	6.23 ± 0.59 fg	5.90 ± 0.47 cd
C11	85.98 ± 0.02 cd	6.23 ± 1.56 fg	3.37 ± 0.41 ab
C12	83.93 ± 0.01 cd	6.70 ± 0.65 defg	3.14 ± 0.04 j
C13	82.91 ± 0.02 de	6.53 ± 0.88 efg	5.96 ± 0.06 d
C14	85.68 ± 0.02 cd	6.53 ± 0.17 efg	3.36 ± 0.07 fgh
C15	76.72 ± 0.00 g	6.90 ± 1.04 cdefg	7.48 ± 0.32 fgh
C16	70.23 ± 0.02 h	10.90 ± 2.33 ab	8.10 ± 0.40 b
C17	86.46 ± 0.03 c	5.30 ± 0.88 g	2.59 ± 0.05 j
C18	85.25 ± 0.02 cd	4.87 ± 0.76 g	2.99 ± 0.08 hij
C19	83.13 ± 0.02 de	9.40 ± 1.36 abcd	5.14 ± 0.07 ij
C20	84.11 ± 0.02 cd	11.03 ± 0.96 ab	5.19 ± 0.05 ghi
C21	79.54 ± 0.03 e	4.27 ± 0.33 g	5.54 ± 0.66 cd
C22	83.24 ± 0.02 de	6.83 ± 1.11 cdefg	3.45 ± 0.16 fghi
C23	83.88 ± 0.02 de	8.53 ± 0.93 bcdef	3.77 ± 0.16 fgh
C24	91.06 ± 0.01 b	6.77 ± 0.86 cdefg	3.61 ± 0.13 c
C25	79.40 ± 0.01 f	10.47 ± 0.17 ab	7.38 ± 0.08 ef
C26	78.85% ± 0.02 g	11.37 ± 0.12 a	7.17 ± 0.06 fg

a−j stands for significant difference.

Table S3. Physiological and biochemical indexes of 26 varieties.

Material Code	Soluble Protein (mg.g⁻¹)	Soluble Sugar (mg.g⁻¹)	Vc (%)	MDA (nmol. g⁻¹)	Relative Conductivity (%)	Cellulose (%)
C1	19.75 ± 0.11 de	11.21 ± 0.08 f	80.18 ± 0.67 de	6.68 ± 0.05 d	18.67 ± 0.01 cd	16.94 ± 1.11 cde
C2	19.83 ± 0.36 de	11.73 ± 0.14 ef	80.20 ± 0.06 de	6.71 ± 0.25 d	17.48 ± 0.01 cde	17.07 ± 0.48 cde
C3	25.00 ± 0.65 b	18.24 ± 0.01 a	81.79 ± 0.44 c	4.73 ± 0.41 e	15.49 ± 0.01 de	26.28 ± 0.98 a
C4	19.60 ± 0.26 de	8.92 ± 0.10 hi	81.70 ± 0.23 c	6.89 ± 0.33 cd	19.38 ± 0.02 c	16.52 ± 0.27 f
C5	23.78 ± 0.32 c	18.39 ± 0.39 a	86.22 ± 0.78 a	4.94 ± 0.45 e	16.47 ± 0.02 cde	8.98 ± 0.49 f
C6	25.13 ± 0.11 b	17.38 ± 0.07 b	83.62 ± 0.14 b	4.96 ± 0.05 e	16.09 ± 0.01 cde	9.40 ± 0.311 f
C7	26.23 ± 0.21 a	16.46 ± 0.31 c	87.17 ± 0.20 a	4.69 ± 0.15 e	14.17 ± 0.01 e	26.94 ± 0.52 a
C8	17.40 ± 0.30 fg	4.58 ± 0.06 j	72.59 ± 0.20 f	9.58 ± 0.63 b	32.66 ± 0.02 ab	8.75 ± 0.26 f
C9	19.42 ± 0.34 de	12.46 ± 0.55 d	79.95 ± 0.94 e	7.59 ± 0.63 b	18.92 ± 0.01 cd	18.00 ± 0.88 c
C10	19.92 ± 0.77 de	12.36 ± 0.10 de	69.56 ± 0.46 g	6.70 ± 0.08 d	18.13 ± 0.01 cde	16.44 ± 0.74 de
C11	19.90 ± 0.12 de	12.89 ± 0.51 d	80.23 ± 0.22 de	6.85 ± 0.08 cd	18.89 ± 0.02 cd	16.92 ± 0.81 cde
C12	19.33 ± 0.51 de	11.70 ± 0.10 ef	80.20 ± 0.07 de	6.96 ± 0.30 cd	18.55 ± 0.01 cd	16.00 ± 0.07 e
C13	19.60 ± 0.47 de	11.49 ± 0.24 f	80.52 ± 0.25 de	7.16 ± 0.29 cd	19.26 ± 0.01 c	16.64 ± 0.12 cde
C14	19.37 ± 0.10 de	12.22 ± 0.09 de	79.62 ± 0.82 e	6.90 ± 0.08 cd	18.64 ± 0.01 cd	16.49 ± 0.28 de
C15	18.16 ± 0.30 f	4.69 ± 0.21 j	68.26 ± 0.10 h	9.93 ± 0.08 b	33.66 ± 0.01 ab	24.21 ± 0.22 b
C16	16.87 ± 0.56 g	4.38 ± 0.10 j	68.24 ± 0.13 h	11.62 ± 0.59 a	36.20 ± 0.01 a	9.62 ± 0.49 f
C17	19.50 ± 0.24 de	9.34 ± 0.07 gh	83.39 ± 0.29 b	5.03 ± 0.38 e	18.72 ± 0.01 cd	24.23 ± 0.77 b
C18	19.96 ± 0.14 de	9.22 ± 0.48 gh	81.31 ± 0.93 cd	6.83 ± 0.09 cd	17.35 ± 0.01 cde	16.86 ± 0.41 cde
C19	20.00 ± 0.07 de	8.36 ± 0.40 i	80.23 ± 0.64 de	6.70 ± 023 d	19.48 ± 0.01 c	16.47 ± 0.64 de
C20	20.25 ± 0.36 d	9.04 ± 0.36 ghi	80.06 ± 0.30 e	6.77 ± 013 d	19.91 ± 0.01 c	17.03 ± 0.72 cde
C21	17.55 ± 0.20 fg	4.70 ± 0.24 j	80.25 ± 0.05 de	10.06 ± 021 b	33.11 ± 0.03 ab	23.37 ± 0.38 b
C22	19.52 ± 0.26 de	11.37 ± 0.08 f	80.23 ± 0.08 de	6.68 ± 0.30 d	18.66 ± 0.02 cd	16.49 ± 0.34 de
C23	19.39 ± 0.56 de	9.71 ± 0.21 g	80.37 ± 0.49 de	6.73 ± 0.12 d	19.14 ± 0.02 cd	17.82 ± 0.64 cd
C24	19.17 ± 0.07 e	12.59 ± 0.18 d	79.64 ± 0.34 e	6.70 ± 0.12 d	19.12 ± 0.02 cd	16.54 ± 0.27

C25	17.58 ± 0.21 fg	4.55 ± 0.11 j	71.92 ± 0.07 f	9.48 ± 0.37 b	31.02 ± 0.01 b	24.73 ± 0.91 b
C26	17.05 ± 0.27 g	4.51 ± 0.51 j	69.43 ± 0.34 g	10.16 ± 0.12 b	31.01 ± 0.02 b	23.58 ± 0.17 b

a−j stands for significant difference.

Table S4. Sequencing data for 12 libraries were obtained by RNA sequencing.

	Varieties	Total Reads	Clean Reads	Clean Bases	Q30	GC	Uniquely Mapped
CK	S16 1	47,115,326	47.12 M	7.06 G	97.20%	48.09%	88.67%
	S16 2	46,773,396	46.77 M	7.00 G	96.85%	47.88%	88.80%
	S16 3	46,879,404	46.88 M	7.02 G	96.91%	48.04%	88.48%
HS	S16 1	46,844,078	46.84 M	7.01 G	97.38%	47.30%	87.70%
	S16 2	47,108,390	47.11 M	7.05 G	97.11%	47.17%	87.97%
	S16 3	47,158,500	47.16 M	7.06 G	96.92%	47.12%	87.63%
CK	J7 1	47,104,308	47.10 M	7.05 G	97.25%	48.12%	87.95%
	J7 2	46,910,192	46.91 M	7.03 G	97.23%	48.32%	88.35%
	J7 3	46,644,998	46.64 M	6.97 G	97.35%	48.68%	87.95%
HS	J7 1	46,503,960	46.50 M	6.96 G	97.20%	47.20%	87.81%
	J7 2	46,069,312	46.07 M	6.90 G	97.21%	46.97%	87.48%
	J7 3	46,768,592	46.77 M	7.00 G	97.23%	47.53%	87.92%

CK and HS represent treatments at 25/18 °C and 43 °C. Q20 and Q30 are the percentage of bases with a Clean Data quality value greater than 20 and 30 as a percentage of the total bases, respectively. GC (%) is the percentage of G and C bases in the total bases in Clean Data. Uniquely mapped is the number of reads aligned to the unique position of the reference genome and its percentage. It should be noted that 1, 2, and 3 represent the three biological repeats from each sample per treatment.

Table S5. List of differentially expressed genes associated with interested pathways under heat stress.

Gene ID	Annotation	Fold Change			
		S16-HS-vs.-S16-CK	J7-HS-vs.-S16-HS	J7-HS-vs.-J7-CK	J7-CK-vs.-S16-CK
Sulfur metabolism					
LOC103860404	APK1	−0.63	1.9	3.72	−2.46
LOC103853800	APK2	−4.38	4.86	2.68	−2.2
LOC103868958	APS1	−0.22	0.54	2.13	−1.82
LOC103847275	SIR	−0.84	0.93	0.43	−0.35
Ribsom					
LOC103849898	RPS9C	6.4	−0.17	8.54	−2.33
LOC103870906	RPS7	1.73	1.21	3.79	−0.85
LOC103830735	ARP2	1.58	0.84	3.7	−1.29
LOC103832205	RPS13	1.72	−0.04	3.65	−2
Glutathione metabolism					
LOC103866350	GSTF8	7.46	0.04	8.51	1.07
LOC103852912	GSTU10	4.01	1.52	6.73	1.2
LOC103872505	GSTU25	2.73	−0.58	4.73	−2.57
LOC103858271	GSTU5	4.57	0.26	5.33	1.03

Table S6. Genes and primers for qRT-PCR analysis.

Gene Name	Primer Name	Primer Sequence (5′->3)
APK1	primer F	GGAAACTCGACGAACATA
	primer R	AGACCAGTGACCCAAATC
SIR	primer F	TAACCGAAGCTCCCAACGTCAA
	primer R	AACTCATCTGCTAAGTCGTCCAT
GSTU25	primer F	GACTCCTCAGCAGATTCAA
	primer R	CCGCCTTCATCATCATCA
GSTU5	primer F	CATCCTCGAGTACATCGACGAGACATG
	primer R	CTGAGCTTCCAGAGGCGTGTCT
HSP70-1	primer F	TACTCTGCTCTCACGCGT
	primer R	CTTGCCTTCAAACTCGTC
HSP90-5	primer F	GTTAGCATGATTGGGCAGTT
	primer R	TCCTTCCTCGTCCTTCTTCT
Actin	primer F	CGAGCTGCGTGTGGCACCTG
	primer R	CATTGCTGGGCAGTTGAAGG

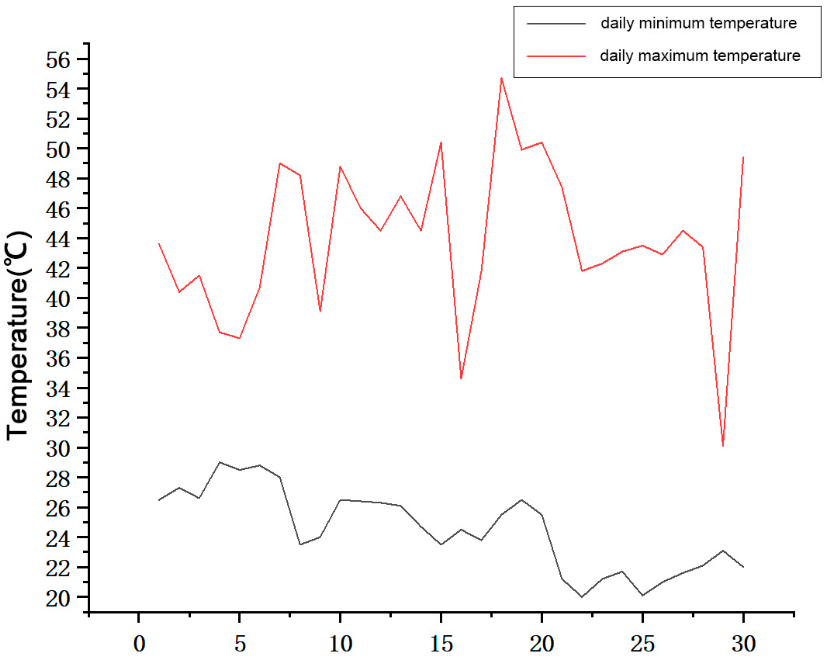

Figure S1. Daily maximum and minimum temperatures change within 30 days of the greenhouse.

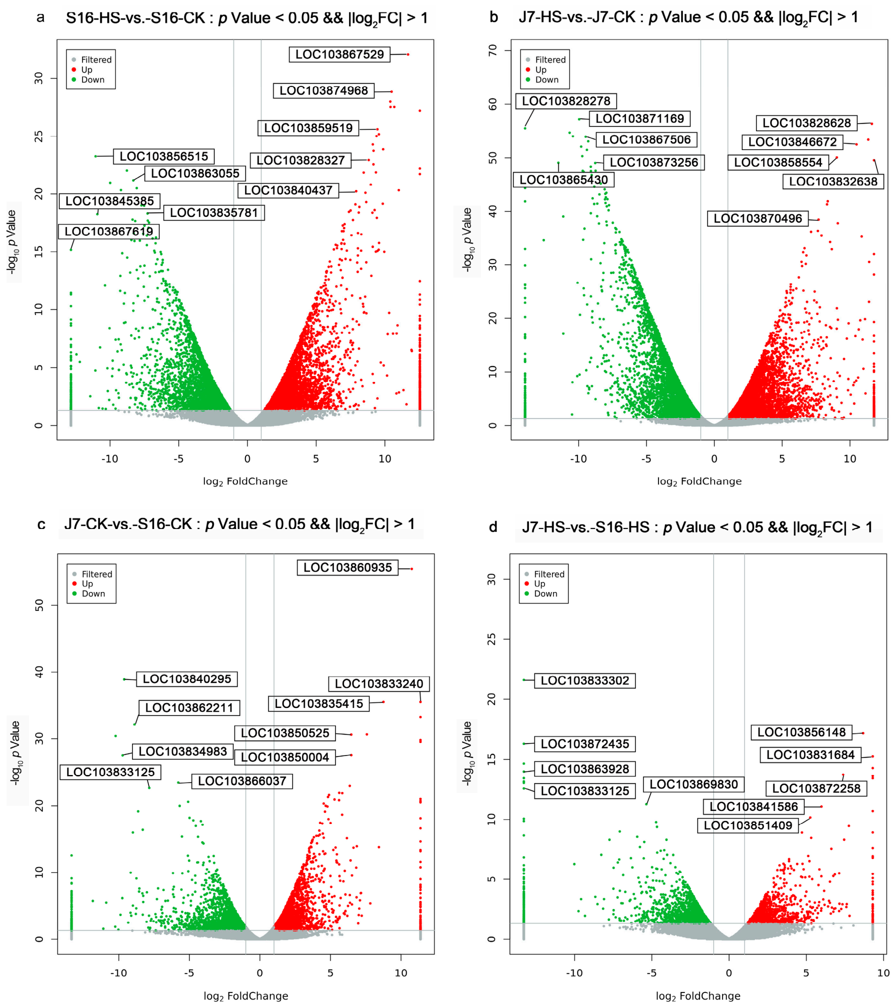

Figure S2. Volcano plots for expressed genes in the four comparison groups. Volcano plots for all the expressed genes in (**a**) S16-HS-vs-S16-CK; (**b**) J7-HS-vs-J7-CK; (**c**) J7-CK-vs-S16-CK; (**d**) J7-HS-vs-S16-HS. The X- and Y-axes present the log2 (FC) for the two samples and −log10 (*p*-value), respectively. Red (upregulated) and green (downregulated) dots indicate that the genes have significant differences, while the gray dots correspond to genes with no significant differences. Each volcano plot was tagged with 10 gene names.

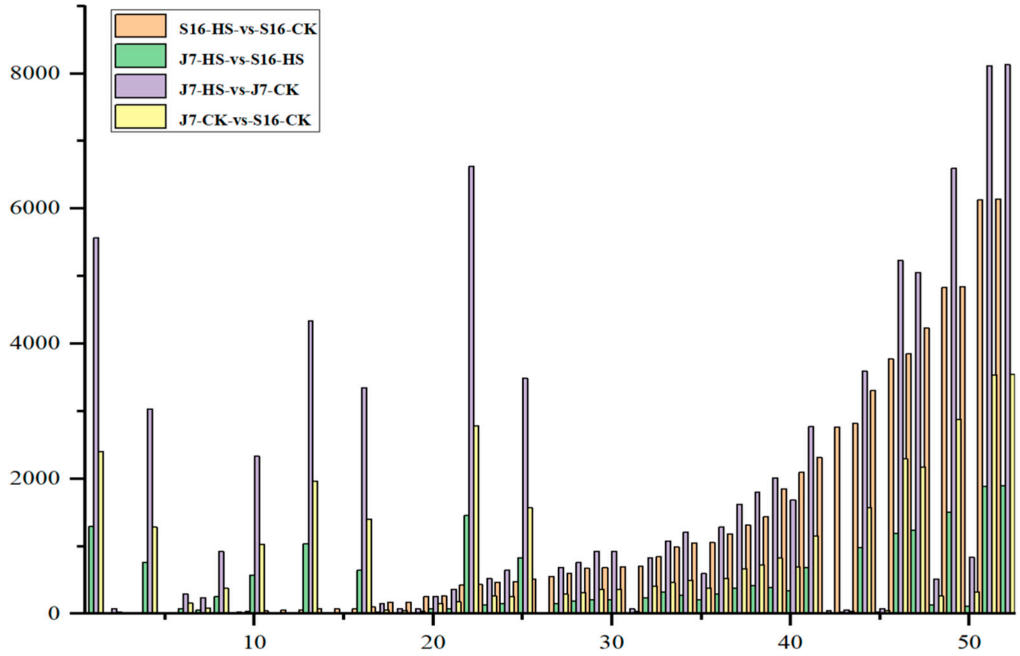

Figure S3. GO enrichment diagram. The Y-axis indicates the number of transcripts, and the X-axis indicates the GO classification. 1: channel regulator activity, 2: protein tag, 3: biological adhesion, 4: biological regulation, 5: cell killing, 6: translation regulator activity, 7: metallochaperone activity, 8: nutrient reservoir activity, 9: locomotion, 10: nucleoid, 11: protein binding transcription factor activity, 12: extracellular region part, 13: electron carrier activity, 14: molecular transducer activity, 15: receptor activity, 16: antioxidant activity, 17: rhythmic process, 18: enzyme regulator activity, 19: structural molecule activity, 20: immune system process, 21: growth, 22: symplast, 23: cell junction, 24: negative regulation of biological process, 25: membrane-enclosed lumen, 26: extracellular region, 27: transporter activity, 28: positive regulation of biological process, 29: reproductive process, 30: reproduction, 31: nucleic acid binding transcription factor activity, 32: multi-organism process, 33: signaling, 34: establishment of localization, 35: macromolecular complex, 36: localization, 37: multicellular organismal process, 38: developmental process, 39: cellular component organization or biogenesis, 40: membrane part, 41: regulation of biological process, 42: organelle part, 43: membrane, 44: response to stimulus, 45: catalytic activity, 46: metabolic process, 47: single-organism process, 48: binding, 49: cellular process, 50: organelle, 51: cell part, 52: cell.

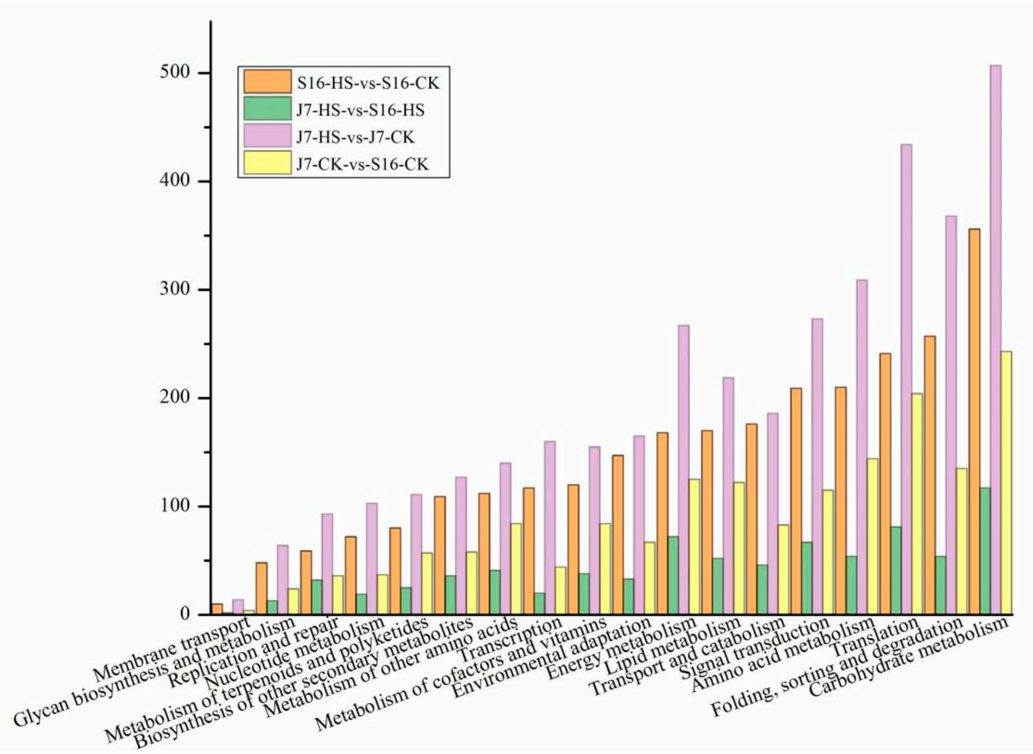

Figure S4. KEGG enrichment diagram.
